# The grasp reflex in patients with idiopathic normal pressure hydrocephalus

**DOI:** 10.1007/s00415-024-12341-0

**Published:** 2024-04-08

**Authors:** Junyan Liu, Shigenori Kanno, Chifumi Iseki, Nobuko Kawakami, Kazuo Kakinuma, Kazuto Katsuse, Shiho Matsubara, Shoko Ota, Keiko Endo, Kentaro Takanami, Shin-ichiro Osawa, Tomohiro Kawaguchi, Hidenori Endo, Shunji Mugikura, Kyoko Suzuki

**Affiliations:** 1 Department of Behavioral Neurology and Cognitive Neuroscience, Tohoku University Graduate School of Medicine, 2-1, Seiryo-machi, Aoba-ku, Sendai, Miyagi 980-8575 Japan; 2 Department of Neurology, The University of Tokyo, Tokyo, Japan; 3 Department of Rehabilitation, Tohoku University Hospital, Sendai, Japan; 4 Department of Diagnostic Radiology, Tohoku University Hospital, Sendai, Japan; 5 Department of Neurosurgery, Tohoku University Graduate School of Medicine, Sendai, Japan; 6 Department of Neurosurgery, Kohnan Hospital, Sendai, Japan; 7 Division of Image Statistics, Tohoku Medical Megabank Organization, Tohoku University, Sendai, Japan

**Keywords:** Attention, Grasp reflex, Frontal lobe, Idiopathic normal pressure hydrocephalus, Primitive reflex

## Abstract

**Objective:**

To investigate the prevalence and intensity of grasp reflexes and to examine changes in these reflexes after shunt surgery in patients with idiopathic normal pressure hydrocephalus (iNPH).

**Methods:**

We enrolled 147 patients with probable iNPH. A standard procedure was used to determine the presence of grasp reflexes, and the intensity of these reflexes was assessed using a four-category classification. Clinical rating scales and their correlation with grasp reflexes were also evaluated. Grasp reflexes were reassessed in 72 patients 1 year after surgery.

**Results:**

We found that approximately 50.3% of patients with iNPH exhibited a positive grasp reflex. Among these patients, 69% exhibited bilateral positivity, while the remaining patients showed unilateral positivity. Furthermore, the intensity of the grasp reflex was significantly correlated with the severity of gait and with cognitive, urinary, motor, and behavioural symptoms. Surgical interventions led to a reduction (41.7%) or maintenance (30.6%) of the reflex intensity in 72.3% of iNPH patients. The changes in reflex intensity showed significant positive correlations with changes in the number of steps of the Timed Up and Go test and Trail Making Test-A scores but not with changes in total scores on the iNPH Grading Scale.

**Conclusion:**

This retrospective study identified grasp reflexes as a highly prevalent phenomenon in patients with iNPH. These reflexes can assist in evaluating the severity of various symptoms, including cognitive, gait, urinary, motor and emotional symptoms.

**Supplementary Information:**

The online version contains supplementary material available at 10.1007/s00415-024-12341-0.

## Introduction

Idiopathic normal pressure hydrocephalus (iNPH) is a progressive syndrome that predominantly occurs among individuals older than the age of 60, with a prevalence ranging from 0.51 to 2.94% in this age group [1]. It is characterized by the clinical triad of gait disturbance, cognitive impairments, and urinary incontinence, with typical brain imaging demonstrating the presence of dilated ventricles, wide Sylvian fissures, and high convexity tightness [2]. Symptoms usually improve after surgical intervention; however, the prognosis is also influenced by factors such as the severity of disease, the timing of the intervention, and comorbidities [3]. Cognitive impairments are dominated by pronounced frontal lobe dysfunctions [3], and gait disorders are also known as frontal gait or higher-level gait disorders [4]. Primitive reflexes, which serve as indicators of frontal lobe dysfunction [5], have the potential to provide objective evidence of these impairments, particularly in individuals exhibiting diminished coordination.

The grasp reflex is a primitive reflex that is defined as the involuntary flexion-adduction movement of the digits in response to distally moving pressure contact applied to a particular area of the palm without any intention to use the object [6, 7]. It is universally present in human foetuses and infants but is suppressed as the central nervous system matures [5, 8, 9]. The recurrence of the grasp reflex in adulthood is linked to localized brain lesions or diffuse neurodegeneration involving the medial frontal lobes and/or their efferent connections, exemplified by anterior cerebral artery infarction and progressive supranuclear palsy (PSP) [10, 11]. However, there is currently no research on the grasp reflex in iNPH patients, despite its prevalence among a large proportion of patients with iNPH in clinical practice.

The grasp reflex reveals a wide range of clinical correlations and potential implications. In Alzheimer's disease (AD), vascular dementia (VaD), and other aetiologies of dementia, the grasp reflex is associated with severe dysfunctions in daily living activities [12, 13], motor dysfunctions, behavioural abnormalities [13, 14], and personality changes [15, 16]. Despite individual exceptions [13, 17], this reflex has also been found to be related to the severity of cognitive dysfunction [14, 18–21]. Moreover, the grasp reflex is associated with urinary symptoms in patients with corticobasal degeneration (CBD) [22] and affects walking gait among preschool children [23]. Another aspect that piques our curiosity is whether these associations persist in iNPH patients and the degree to which their intensity is correlated with other symptoms.

The grasp reflex has been documented as a reversible phenomenon that becomes increasingly difficult to elicit as patients’ conditions improve [24, 25]. Additionally, patients may be able to release the reflex voluntarily after haematoma evacuation or tumour removal surgery [7]. Thomas et al. reported the disappearance of grasp reflexes in two patients with normal pressure hydrocephalus after high-volume CSF removal [26]. However, current publications on the evolution of grasp reflexes are predominantly concentrated in case reports, with few large-sample studies to substantiate their universality.

Therefore, the aims of this study were as follows: (1) to develop a standard procedure for investigating the prevalence, intensity and changes in the grasp reflex in patients with iNPH and (2) to examine whether differences exist between iNPH patients with or without grasp reflexes and to assess the correlation of the grasp reflex with gait, motor skills, cognitive symptoms, urinary symptoms, and behavioural symptoms. We hypothesized that patients with iNPH exhibiting grasp reflexes will exhibit heightened severity of gait, motor, cognitive, urinary and behavioural symptoms, such that the intensity of these symptoms will increase concomitantly with the strength of the grasp reflex. We also hypothesized that surgical intervention can alleviate the intensity of the grasp reflex.

## Materials and methods

### Participants

A total of 147 patients with probable iNPH were retrospectively enrolled in this study from January 2010 to July 2023. All patients underwent neurological and neuropsychological examinations, cranial MRI (*n* = 141), ^123^I-IMP-SPECT (*n* = 144), and CSF tap tests. The diagnosis of iNPH was made by experienced neurologists based on the diagnostic criteria outlined in the Guidelines for Management of Idiopathic Normal Pressure Hydrocephalus [3]. The inclusion criteria for this study were as follows: (1) > 60 years; (2) gait disturbance, cognitive impairment, and/or urinary disturbance; (3) ventricular dilatation on CT/ MRIs (Evans index > 0.3) with features include tight high convexity and medial subarachnoid spaces and enlarged Sylvian fissure (DESH); (4) CSF pressure < 200 mm H_2_O with normal CSF cell counts and protein levels; (5) lack of preceding diseases that may cause ventricular dilatation; and (6) the absence of other diseases that could explain the abovementioned clinical symptoms. The exclusion criteria were as follows: lack of detailed records regarding grasp reflexes; a medical history or comorbidities that could influence the instigation of the grasp reflex, such as frontal lobe infarction, PSP, Parkinson’s disease (PD), or Lewy body dementia; a Blake’s pouch cyst, an arachnoid cyst, or extraventricular CSF accumulation on neuroimaging; or a medical history of psychiatric illness, including schizophrenia or manic depression.

### Evaluation of grasp reflex

#### Inspection methods

The examination was conducted with patients in a seated position. Patients were instructed to open their hands with their fingers naturally flexed downwards. The examiner supported the patients’ wrists on the same side with one hand, avoiding contact with the dorsum of the hand. On the other hand, the examiner placed his palm on the contralateral palm of the patients, positioned between the thumb and index fingers, applying firm pressure on the skin while moving distally from the ulnar to the radial side of the patients' hand and then releasing. A similar movement immediately followed, progressing from the thenar region towards the fingertips. This procedure was performed bilaterally.

#### Scoring criteria

The grasp reflex was considered to be present if there was involuntary closure of the hand. If the reflex was observed, patients were instructed to stop gripping; the sign was rated as ‘2 points’ if patients could successfully inhibit it as instructed and ‘3 points’ if patients were unable to release the grip. If the reflex was not observed, patients were conversed with to divert their attention. If the grasp reflex was observed during this period, ‘1 point’ was assigned; if it was not present throughout, ‘0 points’ were recorded. A score of ‘2 points’ or ‘3 points’ in either hand was considered positive (+), while a score of ‘0 points’ or ‘1 point’ in both hands was considered negative (−). The sum of the scores for both hands constituted the total score, with higher total scores indicating more severe signs. Alternative grouping methods were used, but their use of suboptimal results led to their exclusion (Supplementary Material Fig. 1).

### Evaluation of other neuropsychological symptoms

In all participants, the severity of the triad symptoms was evaluated with the iNPH Grading Scale (iNPHGS) [27]. The degree of disability in daily activities was evaluated with the modified Rankin Scale (mRS) [28]. Mobility and balance were evaluated with the Timed Up and Go test (TUG) [29] and the Functional Balance Scale (FBS) [30]. Motor skills were evaluated with the Movement Disorder Society-Unified Parkinson's Disease Rating Scale (MDS-UPDRS) Part III [31]. General cognitive function was evaluated with the Mini Mental State Examination (MMSE) [32]. Executive function was evaluated with the Frontal Assessment Battery (FAB) and the phonemic and category verbal fluency test (VFT) [33, 34]. In the VFTs, the 1-min free recall of words beginning with each Japanese letter, ‘Fu’, ‘A’, or ‘Ni’ (phonemic), and of animal names (category) was tested. We used the total number of words produced in the phonemic VFTs as the phonemic VFT score. Psychomotor speed was evaluated with the Trail Making Test-A (TMT-A) [35]. Attention and working memory were evaluated with the Wechsler Memory Scale-Revised (WMS-R) Attention/Concentration Index (ACI) and Counting-backward Test (CBT) [36, 37]. Behavioural and psychological symptoms were evaluated with the Neuropsychiatric Inventory (NPI) [38].

### Surgical interventions and follow-up

Twenty-three patients were elderly (approximately 90 years old), had severe comorbidities (heart failure, rectal cancer, etc.), or had high surgical risk (regular use of anticoagulants, recent myocardial infarction, etc.); we recommended shunt treatment for the remaining 124 patients. Twelve patients declined surgery due to concerns about potential adverse events or lack of caregiving. Seventy-eight patients underwent ventriculoperitoneal shunt placement using a Codman-Hakim programmable valve with a Siphon-Guard (Codman and Shurtleff, Integra LifeSciences Corporation, Plainsboro, NJ, USA), and thirty-four patients received lumboperitoneal shunt placement using a Codman-Hakim programmable valve with a Siphon-Guard or a Strata NSC adjustable-pressure valve with a Siphon-Control Device (Medtronic Inc., Minneapolis, MN, USA). All surgical procedures were performed at the Department of Neurosurgery of Tohoku University Hospital or Kohnan Hospital. Postoperatively, if clinical improvement was insufficient, pressure adjustments were made repeatedly until the optimal pressure for the patient was reached. Postoperative subdural haematomas occurred in 9 patients, delirium occurred in 1 patient, and 2 patients underwent revision surgery, all of whom recovered well.

Within the first year postdischarge, 32 patients were lost to follow-up (including 4 who underwent shunt treatment). The reasons for loss to follow-up included the East Japan Great Earthquake, the COVID-19 pandemic, referral to primary care, or transfer for the management of severe comorbidities. One hundred and six patients (including 99 who underwent shunt treatment) were then followed up for a minimum of one year through scheduled clinical evaluation. The remaining 9 patients, who are still within the first year post-surgery, are currently undergoing follow-up. Of the 112 patients who underwent surgery, 72 had detailed data on grasp reflexes at one year postoperatively.

### Statistical analysis

All the statistical analyses were performed using SPSS version 26.0 (IBM Corp., Armonk, NY, USA). For normally distributed quantitative variables, a two-sample t test was used to compare means between groups, while nonnormally distributed quantitative variables were subjected to Mann‒Whitney U tests. Categorical variables were analyzed using chi-square tests. The correlation between the grasp reflex score and other clinical measurements was assessed using Spearman’s rank correlation coefficient and Kendall’s Tau. Paired sample t tests and Wilcoxon signed rank tests were conducted to observe the trends in changes in neuropsychological symptoms from baseline to follow-up. The disparity in the proportion of patients with positive grasp reflexes before and after surgery was examined using the McNemar test. Statistical significance was set at p < 0.05.

## Results

### Demographic and clinical features of the Grasp reflex (+) and Grasp reflex (−) groups

**Fig. 1:**
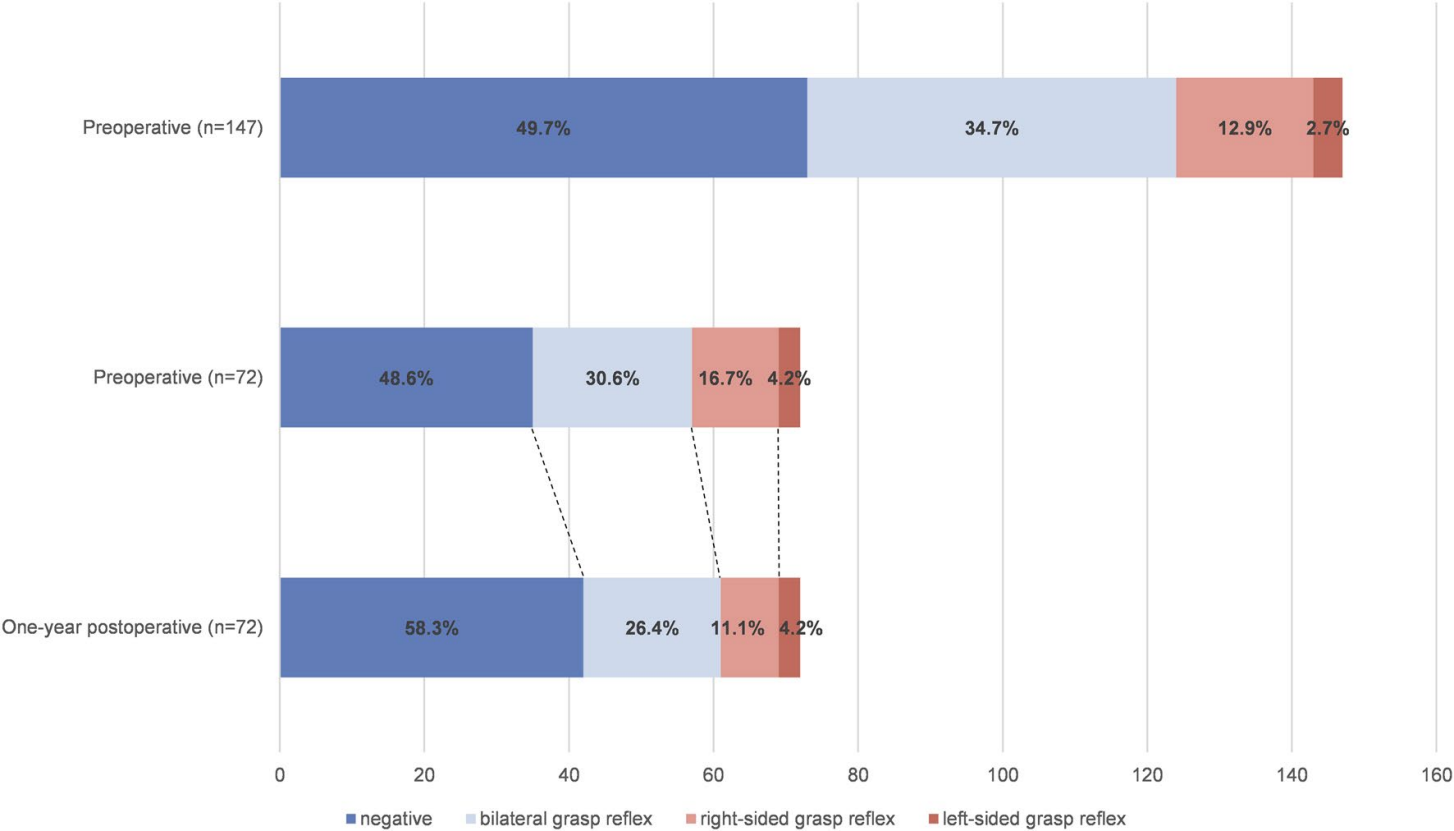
The prevalence and laterality of grasp reflexes in patients with idiopathic normal pressure hydrocephalus. From top to bottom, the presentation illustrates the prevalence and lateral distribution of grasp reflexes for all enrolled patients, as well as the initial and postoperative prevalence and lateral distribution of grasp reflexes in the subgroup followed up 1 year after surgery

Among the 147 patients with iNPH, 50.3% (74/147) were identified as having a positive grasp reflex. Of the 74 patients with a positive grasp reflex, 51 displayed bilateral involvement, 19 showed right-sided involvement, and 4 showed left-sided involvement (Fig. 1). The two groups showed no significant differences in sex, age, disease duration, educational attainment, mRS score, FBS score, or CBT reverse effect index (REI) score. However, compared to those in the Grasp reflex (−) group, patients in the Grasp reflex (+) group exhibited significantly higher scores on the iNPHGS, UPDRS part III, TMT-A, CBT first error score (FES), iNPHGS urination subscale, and NPI, in addition to lower scores on the MMSE, FAB, phonemic and category VFTs, and WMS-R ACI tasks (*p* < 0.05). In the TUG test, the Grasp reflex (+) group took more time (*p* = 0.016), but there was no significant difference in the number of steps between the two groups (*p* = 0.051) (Table 1).

**Table 1:** The demographic and clinical features in the Grasp reflex (+) and Grasp reflex (−) groups

Variables	All	Grasp reflex (+)	Grasp reflex (−)	*p*-value
	*n* = 147	*n* = 74	*n* = 73	
Sex (Male/Female)	77/70	42/32	35/38	0.285
Handiness (Right/Left/Ambidextrous)	140/5/2	72/1/1	68/4/1	–
Age at admission (years)	77.80 (4.68)	78.20 (4.97)	77.40 (4.36)	0.298
Age at onset (years)	73.93 (5.42)	74.58 (5.74)	73.26 (5.02)	0.140
Disease duration (months)	45.00 (26.00, 63.00)	43.00 (23.25, 60.00)	45.00 (27.50, 67.00)	0.184
Education (years)	12.00 (9.00, 12.00)	12.00 (9.00, 12.00)	12.00 (10.50, 13.00)	0.089
iNPHGS total score	6.00 (5.00, 7.00)	6.00 (5.00, 7.00)	5.00 (4.50, 7.00)	**0.003**
mRS score	2.00 (2.00, 3.00)	2.00 (2.00, 3.00)	2.00 (2.00, 3.00)	0.130
TUG [*n* = 129; (+)/(−):64/65]				
Completion time (seconds)	11.60 (9.61, 14.10)	12.50 (9.97, 15.22)	10.40 (9.55, 12.90)	**0.016**
Number of steps	18.00 (15.50, 22.00)	19.00 (16.25, 23.00)	18.00 (15.00, 21.00)	0.051
FBS (/56) [*n* = 139; (+)/(−):68/71]	45.00 (37.00, 50.00)	45.00 (36.25, 49.75)	46.00 (39.00, 50.00)	0.294
UPDRS part III				
Right side [*n* = 76; (+)/(−):33/43]	3.00 (1.00, 6.00)	4.00 (2.00, 6.50)	2.00 (0.00, 4.00)	**0.011**
Left side [*n* = 76; (+)/(−):33/43]	4.00 (1.25, 7.00)	6.00 (3.00, 7.50)	3.00 (0.00, 5.00)	**0.001**
Total (/72) [n = 74; (+)/(−):33/41]	12.00 (9.00, 20.00)	17.00 (12.00, 28.00)	10.00 (6.00, 14.50)	**0.000**
MMSE (/30) [*n* = 146; (+)/(−):73/73]	23.00 (20.00, 25.00)	21.00 (19.00, 24.00)	24.00 (21.50, 26.00)	**0.000**
FAB (/18)	11.00 (8.00, 13.00)	10.00 (7.00, 12.00)	12.00 (9.00, 13.00)	**0.002**
TMT-A (seconds) [*n* = 108; (+)/(−):45/63]	89.50 (61.25, 136.50)	106.00 (80.00, 171.00)	83.00 (57.00, 127.00)	**0.007**
CBT-FES [*n* = 118; (+)/(−):54/64]	2.25 (0.00, 7.75)	4.25 (0.00, 10.00)	1.00 (0.00, 4.25)	**0.033**
CBT-REI [*n* = 104; (+)/(−):41/63]	7.18 (4.05, 10.16)	6.62 (3.92, 11.94)	7.58 (4.76, 9.61)	0.899
PVF (number of words) [*n* = 127; (+)/(−):71/56]	12.00 (8.00, 18.00)	11.00 (7.00, 15.00)	14.00 (10.00, 19.75)	**0.004**
CVF (number of words) [*n* = 129; (+)/(−):70/59]	8.82 (3.67)	7.79 (3.55)	10.05 (3.45)	**0.000**
WMS-R ACI [*n* = 117; (+)/(−):68/49]	78.21 (14.31)	73.96 (15.20)	84.12 (10.54)	**0.000**
iNPHGS urination subscale	1.00 (1.00, 2.00)	1.00 (1.00, 3.00)	1.00 (1.00, 2.00)	**0.015**
NPI total score (severity*frequency, /144) [*n* = 143; (+)/(−):71/72]	9.00 (4.00, 15.00)	9.00 (5.00, 16.00)	7.00 (3.00, 13.00)	**0.022**

The results represent number, means (SD) or medians (p25, p75)

Bold font indicates statistical significance set as a *p* < 0.05

(+), positive; (−), negative; iNPHGS, idiopathic Normal Pressure Hydrocephalus Grading Scale; mRS, modified Rankin Scale; TUG, Timed Up and Go test; FBS, Functional Balance Scale; MDS-UPDRS, Movement Disorder Society-Unified Parkinson’s Disease Rating Scale; MMSE, Mini Mental State Examination; FAB, Frontal Assessment Battery; TMT-A, Trail Making Test-A; CBT, Counting-backward Test; FES, first error score; REI, reverse effect index; PVF, Phonemic Verbal Fluency; CVF, Category Verbal Fluency; WMS-R ACI, Wechsler Memory Scale-Revised Attention/Concentration Index; NPI, Neuropsychiatric Inventory

### Correlations between the grasp reflex score and clinical features

Correlation analyses indicated that the grasp reflex score was significantly positively correlated with iNPHGS total score, mRS score, both the completion time and number of steps of the TUG test, UPDRS part III total score, TMT-A score, CBT-FES score, iNPHGS urination subscale score, and NPI score. Additionally, there were significant negative correlations between the grasp reflex score and the FBS, MMSE, FAB, phonemic and category VFTs, and WMS-R ACI scores. Only the CBT-REI score was not correlated with the grasp reflex score (Table 2).

**Table 2:** Correlations between the grasp reflex score and clinical features

Variables	Grasp reflex score (0–6)	
	*ρ/τ*	*p*-value
iNPHGS total score	0.276	**0.000**
mRS score	0.162	**0.018**
TUG (*n* = 129)		
Completion time (seconds)	0.290	**0.001**
Number of steps	0.268	**0.002**
FBS (/56) (*n* = 139)	− 0.172	**0.043**
UPDRS part III Total (/72) (*n* = 74)	0.453	**0.000**
MMSE (/30) (*n* = 146)	− 0.343	**0.000**
FAB (/18)	− 0.357	**0.000**
TMT-A (seconds) (*n* = 108)	0.340	**0.000**
CBT-FES (*n* = 118)	0.250	**0.006**
CBT-REI (*n* = 104)	0.064	0.520
PVF (number of words) (*n* = 127)	− 0.331	**0.000**
CVF (number of words) (*n* = 129)	− 0.333	**0.000**
WMS-R ACI (*n* = 117)	− 0.449	**0.000**
iNPHGS urination subscale	0.221	**0.001**
NPI total score (*n* = 143) (severity*frequency, /144)	0.280	**0.001**

Bold font indicates statistical significance set at a *p* < 0.05

iNPHGS, idiopathic Normal Pressure Hydrocephalus Grading Scale; mRS, modified Rankin Scale; TUG, Timed Up and Go test; FBS, Functional Balance Scale; MDS-UPDRS, Movement Disorder Society-Unified Parkinson’s Disease Rating Scale; MMSE, Mini Mental State Examination; FAB, Frontal Assessment Battery; TMT-A, Trail Making Test-A; CBT, Counting-backward Test; FES, First error score; REI, Reverse effect index; PVF, Phonemic Verbal Fluency; CVF, Category Verbal Fluency; WMS-R ACI; Wechsler Memory Scale-Revised Attention/Concentration Index; NPI, Neuropsychiatric Inventory

### Changes in grasp reflex after the operation

In the subgroup of 72 patients with postoperative reflex records one year, based on preoperative results, 37 patients were classified into the Grasp reflex (+) group, while 35 patients were classified into the Grasp reflex (−) group.

In both groups, there were significant differences in the iNPHGS total score, the mRS score, both the completion time and the number of steps of the TUG test, the FBS score, and the phonemic VFT and iNPHGS urination subscale scores between baseline and follow-up (*p* < 0.05). However, there were no significant differences in the TMT-A score, CBT-FES score, or WMS-R ACI score between baseline and follow-up. In the Grasp reflex (−) group, there was a significant difference in the UPDRS part III-right side scores (*p* = 0.015) between baseline and follow-up, while the UPDRS part III-left side scores (*p* = 0.058) and total scores (*p* = 0.057) were at the verge of significance. However, none of these variations reached statistical significance in the Grasp reflex (+) group. In the Grasp reflex (+) group, there were significant differences in the MMSE, FAB, CBT-REI, category VFT, and NPI scores between baseline and follow-up (*p* < 0.05). Conversely, no significant differences were found in these variations in the Grasp reflex (−) group (Table 3).

**Table 3:** Changes of clinical features after shunt surgery in the Grasp reflex (+) and Grasp reflex (−) groups

Variables	Baseline	Follow-up	D and 95% CI	*p*-value
iNPHGS total score				
Grasp reflex (+) (*n* = 36)	6.00 (5.00, 7.00)	5.00 (3.25, 6.00)	1.00 (0.50 to 2.00)	**0.000**
Grasp reflex (−) (*n* = 34)	5.00 (5.00, 7.00)	4.00 (3.00, 5.25)	1.50 (1.00 to 2.00)	**0.000**
mRS score				
Grasp reflex (+) (*n* = 36)	2.50 (2.00, 3.00)	2.00 (1.25, 3.00)	0.50 (0.00 to 0.50)	**0.003**
Grasp reflex (−) (*n* = 34)	2.00 (2.00, 3.00)	2.00 (1.00, 2.00)	0.50 (0.00 to 0.50)	**0.006**
TUG				
Completion time (seconds)				
Grasp reflex (+) (*n* = 31)	12.20 (9.66, 14.02)	9.53 (8.70, 11.60)	1.91 (1.18 to 2.87)	**0.000**
Grasp reflex (−) (*n* = 32)	10.42 (8.93, 14.15)	9.10 (7.95, 10.55)	1.96 (0.89 to 3.11)	**0.001**
Number of steps				
Grasp reflex (+) (*n* = 31)	19.00 (16.00, 22.00)	16.00 (14.00, 19.00)	2.50 (1.00 to 4.00)	**0.000**
Grasp reflex (−) (*n* = 32)	17.00 (14.00, 22.75)	15.50 (13.00, 19.00)	2.00 (0.50 to 3.50)	**0.006**
FBS (/56)				
Grasp reflex (+) (*n* = 32)	43.44 (7.72)	46.53 (8.24)	3.09 (0.87 to 5.31)	**0.008**
Grasp reflex (−) (*n* = 32)	44.50 (7.18)	49.44 (5.11)	4.94 (3.14 to 6.74)	**0.000**
UPDRS part III				
Right side				
Grasp reflex (+) (*n* = 19)	4.00 (2.00, 6.00)	5.00 (1.00, 7.00)	0.00 (− 1.50 to 1.50)	0.864
Grasp reflex (−) (*n* = 20)	4.00 (2.00, 7.00)	2.00 (0.25, 4.75)	2.00 (0.00 to 3.50)	**0.015**
Left side				
Grasp reflex (+) (*n* = 19)	5.89 (2.94)	5.84 (3.67)	0.05 (− 1.85 to 1.96)	0.954
Grasp reflex (−) (*n* = 20)	5.30 (4.16)	3.85 (3.03)	1.45 (− 0.06 to 2.96)	0.058
Total (/72)				
Grasp reflex (+) (*n* = 20)	18.90 (8.52)	15.20 (8.95)	3.70 (− 0.37 to 7.77)	0.072
Grasp reflex (−) (*n* = 18)	14.61 (7.08)	11.22 (7.66)	3.39 (− 0.11 to 6.89)	0.057
MMSE (/30)				
Grasp reflex (+) (*n* = 37)	21.30 (4.40)	22.84 (4.57)	1.54 (0.47 to 2.61)	**0.006**
Grasp reflex (−) (*n* = 34)	24.00 (2.98)	24.44 (3.53)	0.44 (− 0.38 to 1.26)	0.282
FAB (/18)				
Grasp reflex (+) (*n* = 37)	10.11 (3.58)	11.32 (3.54)	1.22 (0.37 to 2.06)	**0.006**
Grasp reflex (−) (*n* = 34)	11.32 (2.60)	11.85 (2.70)	0.53 (− 0.42 to 1.48)	0.265
TMT-A (seconds)				
Grasp reflex (+) (*n* = 23)	93.00 (81.00, 150.00)	87.80 (57.00, 163.00)	18.30 (− 23.00 to 51.00)	0.323
Grasp reflex (−) (*n* = 23)	67.00 (52.00, 95.00)	71.00 (55.00, 88.00)	3.55 (− 11.00 to 14.50)	0.648
CBT-FES				
Grasp reflex (+) (*n* = 25)	5.66 (6.82)	5.21 (5.66)	0.45 (− 1.69 to 2.59)	0.667
Grasp reflex (−) (*n* = 26)	3.70 (4.32)	3.06 (3.97)	0.64 (− 0.73 to 2.02)	0.344
CBT-REI				
Grasp reflex (+) (*n* = 20)	5.48 (3.43, 11.34)	4.04 (2.17, 6.18)	1.52 (0.09 to 8.09)	**0.044**
Grasp reflex (−) (*n* = 24)	7.40 (4.25, 8.63)	4.92 (2.99, 9.91)	0.96 (− 0.70 to 2.80)	0.130
PVF (number of words)				
Grasp reflex (+) (*n* = 35)	12.20 (7.26)	15.49 (8.94)	3.29 (0.98 to 5.59)	**0.007**
Grasp reflex (−) (*n* = 27)	13.33 (6.05)	15.85 (6.66)	2.52 (0.18 to 4.86)	**0.036**
CVF (number of words)				
Grasp reflex (+) (*n* = 35)	7.54 (3.31)	10.11 (4.83)	2.57 (1.28 to 3.86)	**0.000**
Grasp reflex (−) (*n* = 28)	9.79 (3.26)	10.39 (4.01)	0.61 (0.63 to 1.84)	0.321
WMS-R ACI				
Grasp reflex (+) (*n* = 35)	76.11 (15.07)	77.00 (15.91)	0.89 (− 1.15 to 2.92)	0.383
Grasp reflex (−) (*n* = 25)	82.24 (9.92)	85.20 (13.08)	2.96 (− 0.64 to 6.56)	0.102
iNPHGS urination subscale				
Grasp reflex (+) (*n* = 36)	1.00 (1.00, 2.75)	1.00 (1.00, 2.00)	0.00 (0.00 to 0.50)	**0.027**
Grasp reflex (−) (*n* = 34)	1.00 (0.75, 2.00)	0.50 (0.00, 1.00)	0.50 (0.00 to 0.50)	**0.004**
NPI total score (severity*frequency, /144)				
Grasp reflex (+) (*n* = 34)	11.65 (6.80)	6.71 (6.12)	4.94 (2.19 to 7.70)	**0.001**
Grasp reflex (−) (*n* = 32)	8.97 (8.15)	7.09 (6.22)	1.88 (− 0.84 to 4.59)	0.169

The results represent means (SD), medians (p25, p75), and Difference and 95% Confidence Interval

Bold font indicates statistical significance set as a *p* < 0.05

(+), positive; (−), negative; iNPHGS, idiopathic Normal Pressure Hydrocephalus Grading Scale; mRS, modified Rankin Scale; TUG, Timed Up and Go test; FBS, Functional Balance Scale; MDS-UPDRS, Movement Disorder Society-Unified Parkinson’s Disease Rating Scale; MMSE, Mini Mental State Examination; FAB, Frontal Assessment Battery; TMT-A, Trail Making Test-A; CBT, Counting-backward Test; FES, First error score, REI, Reverse effect index; PVF, Phonemic Verbal Fluency; CVF, Category Verbal Fluency; WMS-R ACI, Wechsler Memory Scale-Revised Attention/Concentration Index; NPI, Neuropsychiatric Inventory

The postoperative proportion of patients identified as Grasp reflex (+) decreased from 51.4% (37/72) to 41.7% (30/72). Specifically, within the cohort of 30 individuals with positive grasp reflexes, 19 presented with bilateral involvement, 8 exhibited right-sided involvement, and 3 exhibited left-sided involvement (Fig. 1). The difference in the proportion of patients who were positive before surgery compared with after surgery did not reach statistical significance (*p* = 0.265). In terms of reflex intensity, 41.7% (30/72) of patients exhibited a mitigated grasp reflex, 30.6% (22/72) showed no change, and the remainder experienced an exacerbation. Furthermore, there were significant positive correlations between changes in grasp reflex intensity and changes in the number of steps of the TUG test, UPDRS part III total score, and TMT-A (*p* < 0.05). After controlling for the effects of disease duration and severity (iNPHGS total scores), the correlation between changes in reflex intensity and UPDRS part III total scores was found to be nonsignificant (*p* = 0.055). No correlations were found between changes in reflex intensity and changes in iNPHGS total score (*p* = 0.411) (Supplementary material Table 1).

## Discussion

This is the first study to investigate the grasp reflex in iNPH patients. We elucidated that approximately 50.3% of patients with iNPH exhibited a positive grasp reflex, with a bilateral predominance. Furthermore, the intensity of the grasp reflex was significantly correlated with the severity of gait as well as with cognitive, urinary, motor, and behavioural symptoms. Surgical interventions led to a reduction or maintenance of the reflex intensity in 72.3% of iNPH patients. Changes in reflex intensity were correlated with changes in stride length and psychomotor speed, but no correlation was observed with changes in iNPHGS total scores.

Previous studies describing grasp reflexes have covered ‘weak, moderate, and strong’, ‘complete or incomplete closure’, and ‘persistent or nonpersistent contractions’ [7, 24, 39]. We believe that specifying the degree of these responses may be challenging for nonexamining physicians. Hence, we adopted a more easily quantifiable four-category classification system for grasp reflexes: absent (0 points), elicited under distraction (1 point), elicited but suppressible (2 points), and elicited and nonsuppressible (3 points). In fully conscious patients, significant modifications related to varying degrees of patient attention are observed in the grasp reflex. In general, grasp reflexes are more easily triggered when the subject’s attention is diverted. Moreover, the ability to release reflexes is not only associated with training effects and extensor muscle strength but also closely related to the capacity to concentrate [7, 40]. In our study, patients exhibiting reflexes only when disturbed were assigned a score of 1. Remarkably, most patients could voluntarily relax the reflex. Conversely, patients who were unable to cease grasping despite commands scored 3 points, thus highlighting ineffective attention concentration. Our scoring method reflected an increasing impairment of attention in patients. On the other hand, the significant correlation between grasp reflex and both the WMS-R ACI score, as well as the CBT-FES score, also supported this observation.

In healthy adults and elderly individuals, the prevalence of grasp reflexes typically falls within the range of 0% to 5.88% [12, 21, 41–45]. In practice, our method never elicits a reflex in healthy elderly individuals. In patients with localized brain injury, the prevalence of grasp reflexes ranges from 8 to 18% [46, 47]. If the injury is an anterior cerebral artery infarction or lacunar infarct, the prevalence of the grasp reflex will increase to 25–40% [10, 45, 48, 49]. In AD, VaD, and other aetiologies of dementia, the prevalence of grasp reflexes ranges from 0% to 33.9% (the respective prevalences are shown in Table 4) [11, 17, 19, 50–53]. Importantly, two studies reported a 50% probability among patients with PD and patients with CBD. However, both studies had small sample sizes—only 8 patients with PD [20] and 10 patients with CBD [22]. This raises concerns about potential problems due to the small sample size. Even when restricting our analysis to patients able to elicit the reflex under focused attention, our study identified abnormalities in 50.3% of the patients. Therefore, we can conclude that the grasp reflex is a relatively common phenomenon in patients with iNPH.

**Table 4:** The prevalence of grasp reflexes in various disease

Disease	Prevalence
AD	0–33.9%
VaD	0–21%
PD and PDD	0–50%
PSP	0–19%
CBD	0–50%
LBD	6.25–15%
FTD	0–4.17%

AD, Alzheimer’s disease; VaD, vascular dementia; PD and PDD: Parkinson’s disease and Parkinson’s disease dementia; PSP, progressive supranuclear palsy; CBD, corticobasal degeneration; LBD, Lewy body dementia; FTD, frontotemporal dementia

Research has indicated that a unilateral grasp reflex suggests damage to the contralateral frontal lobe [47], while a bilateral grasp reflex lacks specific localizing value and is often associated with generalized and vague lesions [54]. The grasp reflex in most patients with focal brain injuries is bilateral or contralateral to the lesion. In CBD, grasp reflexes commonly appear unilaterally, aligning with the strikingly asymmetric features of CBD [22, 55, 56]. In instances where grasp reflexes are positive in patients with AD, PD, or VaD, the probabilities of bilateral reflexes are 76–94%, 82%, and 51%, respectively [12, 57]. This is consistent with the diffuse or focal nature of lesions characteristic of each disease. Among our positive patients, 69% displayed positivity on both sides, and the remaining patients exhibited unilateral positivity. In addition, 36% (53/147) of patients showed varying reflex intensities on both sides. Three reasons may be associated with the asymmetry observed in patients with iNPH. First, as the grasp reflex is considered a release sign indicating cortical disinhibition, this may suggest asymmetric cortical disinhibition. Second, this difference could be attributed to the distribution of periventricular or subcortical white matter lesions. Third, asymmetrical ventricular enlargement may also contribute to this phenomenon.

However, the pathophysiologic basis of grasp reflexes in iNPH patients remains unclear. In patients with AD and subcortical infarction, the appearance of grasp reflexes has been found to be associated with ventricular enlargement [18, 49]. Schuster and Casper proposed a hypothesis (cited by Bucy and De Renzi): there exists a hypothetical inhibitory pathway originating from the bilateral medial surface of the superior frontal convolution and of the cingulate gyrus, descending through the white matter anterior and lateral to the frontal horn before leading towards the bilateral central area. The extreme degree of hydrocephalus caused compression of the occipito-frontal fasciculus region by the superolateral angle of the lateral ventricles or a marked increase in intracranial tension, both of which may result in the grasp reflex [46, 54]. We speculate that patients with iNPH may conform to the abovementioned hypothesis.

Odenheimer et al. [58] reported that the incidence of grasp reflexes increases with age. However, our study, in line with others [13, 14, 59], suggested that the distribution of grasp reflexes is not affected by age. In Burn et al.'s research, the presence of grasp reflexes in AD patients was associated with a younger age of onset and a longer disease duration [18]; these findings were not corroborated in our study. This disparity may be due to the different probabilities of patients exhibiting grasp reflexes between the two studies. In their research, this probability was 7.3% (13/161), whereas in our study, it was 50.3% (74/147).

Our study revealed that patients in the positive control group exhibited higher iNPHGS scores than patients in the negative control group, and the reflex intensity was positively correlated with those scores. In PD, grasp reflexes are exclusively observed in Hoehn and Yahr stage III and IV patients [60]. They have also been found specifically in AD patients with Global Deterioration Scale stages 6 and 7 [61, 62]. These findings suggest that the grasp reflex is sensitive to disease severity in iNPH patients. Moreover, although there was no statistically significant difference in mRS scores between the two groups in our study, there was an increasing trend in the correlation with increased reflex scores. Studies have indicated that individuals with dementia who exhibit grasp reflexes often experience more functional problems [12, 13], and with the deterioration of function, the mean activity rating of the reflex also tends to increase [63]. The observed discrepancies may arise from the inherent imprecision of our measurement scale. Moreover, despite greater disease severity possibly being the primary explanation for functional impairments, we found that grasp reflex intensity reliably reflects the extent of functional impairments in iNPH patients.

In a cohort of 201 dementia patients with grasp reflexes, the prevalence of gait, balance, posture, and tone abnormalities was 89.6%, 89.0%, 83.1%, and 72.1%, respectively. Moreover, 28.8% of the patients displayed bradykinesia [12]. Alterations in grasp reflexes have been observed in conjunction with gradual deterioration in both gait and posture among AD patients [62]. In our study, the positive group demonstrated prolonged completion time in the TUG test and higher UPDRS part III scores, and both of these variables were correlated with the intensity of the grasp reflex. Despite the lack of significant differences in the number of steps during the TUG test or in the FBS values, they still exhibited a correlation with reflex intensity. Furthermore, there was no significant improvement in the UPDRS part III scores of patients in the positive group after surgery. Studies have suggested that gait disturbances in patients with iNPH are linked to disrupted connectivity between the supplementary motor area and subcortical structures, as well as insufficient inhibitory control from the premotor and subcortical regions [64, 65]. Furthermore, striatal dopaminergic dysfunction serves as the pathophysiological basis for gait disturbances and parkinsonian signs [66]. The intersection between these regions and areas responsible for inhibiting the grasp reflex can explain the correlation between them [67, 68].

Many studies of dementia patients have consistently demonstrated that individuals with grasp reflexes exhibit more profound cognitive dysfunction [12, 14, 18, 20, 21, 40, 62], and our patients with iNPH are no exception to this pattern. Moreover, our study demonstrated a more comprehensive relationship, indicating close correlations between grasp reflexes and executive function, psychomotor speed, working memory, and attention. On the other hand, Simpson et al. demonstrated that grasp reflexes are associated with depressive symptoms in patients with vascular dementia [16]. Our study also indicated that patients with grasp reflexes present more neuropsychiatric issues. Due to the strong correlation between grasp reflexes and prefrontal cortical functions such as cognition, emotion, and memory [67], we consider the grasp reflex to be an adjunctive tool for assessing cognitive-behavioural impairments in patients with iNPH. Given the greater postoperative cognitive improvement observed in the positive group, we hypothesize that this phenomenon may be attributed to their inferior baseline performance.

In AD patients, the prevalence of grasp reflexes has been found to be more than twice as high in those with permanent double incontinence than in those with incipient incontinence and approximately 11 times greater than that in continent individuals [69]. Our findings are consistent with the results of previous studies, indicating that patients with positive grasp reflexes exhibit more severe urinary problems. The overlap between the cortical mechanisms controlling the micturition reflex and mediating the perception of bladder distension and those inhibiting the grasp reflex forms the basis for the correlation between the two [70–72].

Our study represents the first investigation of reflex changes based on a large sample size. In our study, a reduction in reflex intensity was observed in 41.7% of patients following surgical intervention; however, overall, this improvement did not reach statistical significance at the group level. Lenfeldt et al. reported that in patients with iNPH, significant improvements in motor performance following CSF drainage were accompanied by enhanced activation in supplementary motor areas [73]. This mechanism may also be attributed to the neural substrate underlying the reduction in reflex intensity in our patients. Additionally, our study revealed significant correlations between reductions in reflex intensity and improvements in the number of steps of the TUG test and TMT-A. However, further validation with a larger sample size is necessary to determine whether changes in reflex intensity can accurately predict postoperative alterations in stride length and psychomotor speed. Furthermore, the changes in reflex intensity showed no significant correlation with changes in iNPHGS total scores, indicating that grasp reflexes do not have predictive value for postoperative outcomes, contrary to the hypothesis proposed by Thomas et al. [26].

This study has five main limitations. First, this study did not include a healthy or disease control group. Second, the retrospective review of clinical records employed in this study is susceptible to errors stemming from variations among examiners in technique or the reliability of documenting abnormalities. Third, the scoring method and classification used were not validated. Different assessment methods and scoring criteria may lead to disparate prevalence rates across various studies. Fourth, this manuscript lacked the inclusion of imaging findings and examinations of AD biomarkers, which could better elucidate the underlying anatomical and pathophysiological mechanisms of the grasp reflex in iNPH patients. Fifth, in the analysis of changes in the grasp reflex after the operation, we were unable to obtain reflex data at one year postoperatively or beyond for all patients who underwent surgery due to the limitations of retrospective research. Nevertheless, this retrospective study was rooted in substantive observations from more than a decade of clinical practice. We did not observe such widespread grasp reflexes in healthy ageing or in patients with other neurodegenerative diseases. Additionally, each clinical practitioner underwent rigorous training before assuming their position. Furthermore, we are actively engaged in a prospective validation study aimed at validating the feasibility of our methodology. Finally, we have been conducting studies on the grasp reflex based on various neuroimaging techniques with the aim of providing valuable insights into the mechanisms underlying the grasp reflex in iNPH patients. Prospective studies on the comorbidity of iNPH and AD are also ongoing.

## Conclusion

Our study identified grasp reflexes as a highly prevalent phenomenon in patients with iNPH. This reflex can assist in evaluating the severity of various symptoms, including cognitive, gait, urinary, motor and emotional symptoms. Notably, it is often overlooked in routine neurological examinations at most small- to medium-sized hospitals, leading to a gradual loss of attention. This article aimed to increase awareness and emphasize the significance of the grasp reflex. Future research can focus on white matter lesions associated with grasp reflexes, a phenomenon that currently remains unclear.

### Supplementary Information

Below is the link to the electronic supplementary material.

Supplementary file1 (DOCX 134 kb)

## Data Availability

The datasets used and/or analysed during the current study are not publicly available due to participant’s privacy protections but are available from the corresponding author upon reasonable request.

## References

[1] Martín-Láez R, Caballero-Arzapalo H, López-Menéndez L (2015). Epidemiology of idiopathic normal pressure hydrocephalus: a systematic review of the literature. World Neurosurg.

[2] Wang Z, Zhang Y, Hu F (2020). Pathogenesis and pathophysiology of idiopathic normal pressure hydrocephalus. CNS Neurosci Ther.

[3] Nakajima M, Yamada S, Miyajima M (2021). Guidelines for management of idiopathic normal pressure hydrocephalus (third edition): endorsed by the Japanese Society of Normal Pressure Hydrocephalus. Neurol Med Chir (Tokyo).

[4] Nutt JG (2013). Higher-level gait disorders: an open frontier. Mov Disord.

[5] Schott JM, Rossor MN (2003). The grasp and other primitive reflexes. J Neurol Neurosurg Psychiatry.

[6] Thomas RJ (1994). Blinking and the release reflexes: are they clinically useful?. J Am Geriatr Soc.

[7] Seyffarth H, Denny-Brown D (1948). The grasp reflex and the instinctive grasp reaction. Brain.

[8] Jakobovits AA (2009). Grasping activity in utero: a significant indicator of fetal behavior (the role of the grasping reflex in fetal ethology). J Perinat Med.

[9] Mestre T, Lang AE (2010). The grasp reflex: a symptom in need of treatment. Mov Disord.

[10] Kang SY, Kim JS (2008). Anterior cerebral artery infarction: stroke mechanism and clinical-imaging study in 100 patients. Neurology.

[11] Cordato NJ, Halliday GM, Caine D (2006). Comparison of motor, cognitive, and behavioral features in progressive supranuclear palsy and Parkinson's disease. Mov Disord.

[12] Hogan DB, Ebly EM (1995). Primitive reflexes and dementia: results from the Canadian Study of Health and Aging. Age Ageing.

[13] Molloy DW, Clarnette RM, Mcilroy WE (1991). Clinical significance of primitive reflexes in Alzheimer's disease. J Am Geriatr Soc.

[14] Bakchine S, Lacomblez L, Palisson E (1989). Relationship between primitive reflexes, extra-pyramidal signs, reflective apraxia and severity of cognitive impairment in dementia of the Alzheimer type. Acta Neurol Scand.

[15] Aitken L, Simpson S, Burns A (1999). Personality change in dementia. Int Psychogeriatr.

[16] Simpson S, Allen H, Tomenson B (1999). Neurological correlates of depressive symptoms in Alzheimer's disease and vascular dementia. J Affect Disord.

[17] Marterer-Travniczek A, Danielczyk W, Müller F (1992). Release signs in Parkinson's disease with and without dementia. J Neural Transm Park Dis Dement Sect.

[18] Burns A, Jacoby R, Levy R (1991). Neurological signs in Alzheimer's disease. Age Ageing.

[19] Förstl H, Burns A, Levy R (1992). Neurologic signs in Alzheimer's disease. Results of a prospective clinical and neuropathologic study. Arch Neurol.

[20] Tweedy J, Reding M, Garcia C (1982). Significance of cortical disinhibition signs. Neurology.

[21] Galasko D, Kwo-on-Yuen PF, Klauber MR (1990). Neurological findings in Alzheimer's disease and normal aging. Arch Neurol.

[22] Sakakibara R, Uchiyama T, Yamanishi T (2004). Urinary function in patients with corticobasal degeneration; comparison with normal subjects. Neurourol Urodyn.

[23] Gieysztor E, Kowal M, Paprocka-Borowicz M (2022). Primitive reflex factors influence walking gait in young children: an observational study. Int J Environ Res Public Health.

[24] Adie WJ, Critchley M (1927). Forced Grasping and Groping. Brain.

[25] Shahani B, Burrows PT, Whitty CW (1970). The grasp reflex and perseveration. Brain.

[26] Thomas RH, Bennetto L, Silva MT (2009). Reversible grasp reflexes in normal pressure hydrocephalus. Clin Neurol Neurosurg.

[27] Kubo Y, Kazui H, Yoshida T (2008). Validation of grading scale for evaluating symptoms of idiopathic normal-pressure hydrocephalus. Dement Geriatr Cogn Disord.

[28] Broderick JP, Adeoye O, Elm J (2017). Evolution of the modified Rankin Scale and its use in future stroke trials. Stroke.

[29] Podsiadlo D, Richardson S (1991). The timed "Up & Go": a test of basic functional mobility for frail elderly persons. J Am Geriatr Soc.

[30] Muir SW, Berg K, Chesworth B (2008). Use of the Berg Balance Scale for predicting multiple falls in community-dwelling elderly people: a prospective study. Phys Ther.

[31] Goetz CG, Tilley BC, Shaftman SR (2008). Movement Disorder Society-sponsored revision of the Unified Parkinson's Disease Rating Scale (MDS-UPDRS): scale presentation and clinimetric testing results. Mov Disord.

[32] Folstein MF, Folstein SE, Mchugh PR (1975). "Mini-mental state". A practical method for grading the cognitive state of patients for the clinician. J Psychiatr Res.

[33] Dubois B, Slachevsky A, Litvan I (2000). The FAB: a Frontal Assessment Battery at bedside. Neurology.

[34] Shao Z, Janse E, Visser K (2014). What do verbal fluency tasks measure? Predictors of verbal fluency performance in older adults. Front Psychol.

[35] Tombaugh TN (2004). Trail Making Test A and B: normative data stratified by age and education. Arch Clin Neuropsychol.

[36] Loring DW (1989). The Wechsler memory scale-revised, or the Wechsler memory scale-revisited?. Clin Neuropsychol.

[37] Kanno S, Saito M, Hayashi A (2012). Counting-backward test for executive function in idiopathic normal pressure hydrocephalus. Acta Neurol Scand.

[38] Cummings JL (1997). The neuropsychiatric inventory. Neurology.

[39] Vreeling FW, Jolles J, Verhey FR (1993). Primitive reflexes in healthy, adult volunteers and neurological patients: methodological issues. J Neurol.

[40] Jenkyn LR, Walsh DB, Culver CM (1977). Clinical signs in diffuse cerebral dysfunction. J Neurol Neurosurg Psychiatry.

[41] Brown DL, Smith TL, Knepper LE (1998). Evaluation of five primitive reflexes in 240 young adults. Neurology.

[42] Kokmen E, Bossemeyer RW, Barney J (1977). Neurological manifestations of aging. J Gerontol.

[43] Benassi G, D'alessandro R, Gallassi R (1990). Neurological examination in subjects over 65 years: an epidemiological survey. Neuroepidemiology.

[44] Van Boxtel MP, Bosma H, Jolles J (2006). Prevalence of primitive reflexes and the relationship with cognitive change in healthy adults: a report from the Maastricht Aging Study. J Neurol.

[45] Rao R, Jackson S, Howard R (1999). Primitive reflexes in cerebrovascular disease: a community study of older people with stroke and carotid stenosis. Int J Geriatr Psychiatry.

[46] De Renzi E, Barbieri C (1992). The incidence of the grasp reflex following hemispheric lesion and its relation to frontal damage. Brain.

[47] Paulson G, Gottlieb G (1968). Development reflexes: the reappearance of foetal and neonatal reflexes in aged patients. Brain.

[48] Ishii N, Nishihara Y, Imamura T (1986). Why do frontal lobe symptoms predominate in vascular dementia with lacunes?. Neurology.

[49] Corbett AJ, Bennett H, Kos S (1992). Frontal signs following subcortical infarction. Clin Exp Neurol.

[50] Borroni B, Broli M, Costanzi C (2006). Primitive reflex evaluation in the clinical assessment of extrapyramidal syndromes. Eur J Neurol.

[51] Di Legge S, Di Piero V, Altieri M (2001). Usefulness of primitive reflexes in demented and non-demented cerebrovascular patients in daily clinical practice. Eur Neurol.

[52] Links KA, Merims D, Binns MA (2010). Prevalence of primitive reflexes and Parkinsonian signs in dementia. Can J Neurol Sci.

[53] Diehl-Schmid J, Schūlte-Overberg J, Hartmann J (2007). Extrapyramidal signs, primitive reflexes and incontinence in fronto-temporal dementia. Eur J Neurol.

[54] Bucy PC (1931). Reflex-grasping associated with tumours not involving the frontal lobes. Brain.

[55] Riley DE, Lang AE, Lewis A (1990). Cortical-basal ganglionic degeneration. Neurology.

[56] Doody RS, Jankovic J (1992). The alien hand and related signs. J Neurol Neurosurg Psychiatry.

[57] Basavaraju NG, Silverstone FA, Libow LS (1981). Primitive reflexes and perceptual sensory tests in the elderly- their usefulness in dementia. J Chronic Dis.

[58] Odenheimer G, Funkenstein HH, Beckett L (1994). Comparison of neurologic changes in 'successfully aging' persons vs the total aging population. Arch Neurol.

[59] Huff FJ, Growdon JH (1986). Neurological abnormalities associated with severity of dementia in Alzheimer's disease. Can J Neurol Sci.

[60] Huber SJ, Paulson GW (1986). Relationship between primitive reflexes and severity in Parkinson's disease. J Neurol Neurosurg Psychiatry.

[61] Franssen EH, Reisberg B, Kluger A (1991). Cognition-independent neurologic symptoms in normal aging and probable Alzheimer's disease. Arch Neurol.

[62] Franssen EH, Reisberg B (1997). Neurologic markers of the progression of Alzheimer's disease. Int Psychogeriatr.

[63] Franssen EH, Kluger A, Torossian CL (1993). The neurologic syndrome of severe Alzheimer's disease. Relationship to functional decline. Arch Neurol.

[64] Chistyakov AV, Hafner H, Sinai A (2012). Motor cortex disinhibition in normal-pressure hydrocephalus. J Neurosurg.

[65] Kanno S, Abe N, Saito M (2011). White matter involvement in idiopathic normal pressure hydrocephalus: a voxel-based diffusion tensor imaging study. J Neurol.

[66] Todisco M, Zangaglia R, Minafra B (2021). Clinical outcome and striatal dopaminergic function after shunt surgery in patients with idiopathic normal pressure hydrocephalus. Neurology.

[67] Thompson AE, Thompson PD (2023). Frontal lobe motor syndromes. Handb Clin Neurol.

[68] Choi I, Jung KI, Yoo WK (2015). Novel information on anatomic factors causing grasp reflex in frontal lobe infarction: a case report. Ann Rehabil Med.

[69] Franssen EH, Souren LE, Torossian CL (1997). Utility of developmental reflexes in the differential diagnosis and prognosis of incontinence in Alzheimer's disease. J Geriatr Psychiatry Neurol.

[70] Andrew J, Nathan PW (1964). Lesions on the anterior frontal lobes and disturbances of micturition and defaecation. Brain.

[71] Sakakibara R, Uchida Y, Ishii K (2012). Correlation of right frontal hypoperfusion and urinary dysfunction in iNPH: a SPECT study. Neurourol Urodyn.

[72] Sasaki H, Ishii K, Kono AK (2007). Cerebral perfusion pattern of idiopathic normal pressure hydrocephalus studied by SPECT and statistical brain mapping. Ann Nucl Med.

[73] Lenfeldt N, Larsson A, Nyberg L (2008). Idiopathic normal pressure hydrocephalus: increased supplementary motor activity accounts for improvement after CSF drainage. Brain.

