# Supplementary material for: The grasp reflex in patients with idiopathic normal pressure hydrocephalus
Source: J Neurol. 2024 Apr 8;271(7):4191–202. doi: 10.1007/s00415-024-12341-0 (PMC11233324; doi:10.1007/s00415-024-12341-0)
Supplement: Supplementary file 1 — Supplementary file1 (DOCX 134 kb) [file 415_2024_12341_MOESM1_ESM.docx]

**Supplementary material**


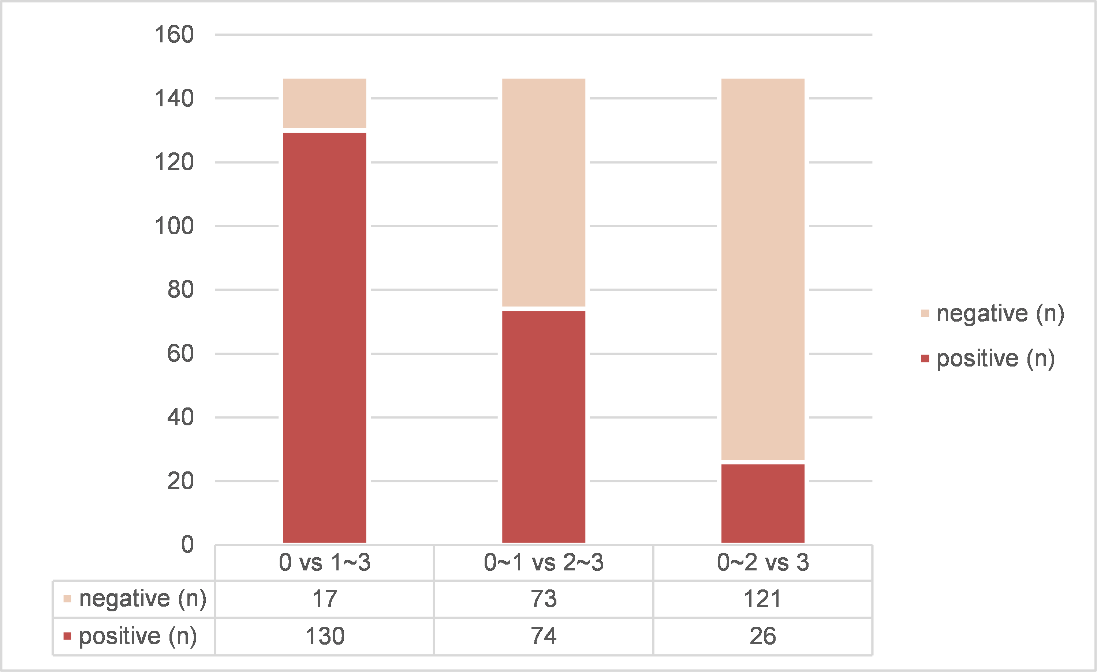


Fig. 1 Alternative grouping methods of grasp reflexes in patients with idiopathic Normal Pressure Hydrocephalus. From left to right, the distribution patterns of positive reflexes are sequentially presented based on categorizations of 0 scores against 1 to 3 scores, 0 to 1 scores against 2 to 3 scores, and 0 to 2 scores against 3 scores. n: numbers.

Table 1 Correlations between changes in both grasp reflex scores and clinical features after surgery

|  | Changes in grasp reflex scores | | Adjusted | |
| --- | --- | --- | --- | --- |
| Changes in variables | ρ | P-value | ρ | P-value |
| iNPHGS total score (n=70) | 0.120 | 0.324 | 0.101 | 0.411 |
| mRS score (n=70) | 0.029 | 0.811 | 0.039 | 0.754 |
| TUG (n=63) |  |  |  |  |
| Completion time (seconds) | 0.124 | 0.333 | 0.046 | 0.726 |
| Numbers of steps | 0.324 | **0.010** | 0.303 | **0.019** |
| FBS (/56) (n=64) | 0.005 | 0.970 | 0.055 | 0.675 |
| UPDRS part III Total (/72) (n=38) | 0.322 | **0.049** | 0.323 | 0.055 |
| MMSE (/30) (n=71) | 0.001 | 0.996 | 0.070 | 0.571 |
| FAB (/18) (n=71) | -0.043 | 0.722 | -0.039 | 0.752 |
| TMT-A (seconds) (n=46) | 0.339 | **0.021** | 0.320 | **0.037** |
| CBT-FES (n=51) | 0.035 | 0.805 | 0.065 | 0.661 |
| CBT-REI (n=44) | 0.002 | 0.990 | -0.084 | 0.601 |
| PVF (numbers of words) (n=62) | -0.078 | 0.549 | -0.076 | 0.567 |
| CVF (numbers of words) (n=63) | -0.214 | 0.093 | -0.189 | 0.149 |
| WMS-R ACI (n=60) | -0.159 | 0.226 | -0.215 | 0.108 |
| iNPHGS urination subscale (n=70) | -0.024 | 0.845 | -0.068 | 0.582 |
| NPI total score (n=66) (severity*frequency, /144) | 0.057 | 0.647 | 0.028 | 0.827 |

Bold font indicates statistical significance set as a p<0.05.

The correlation between the changes in both grasp reflex scores and other clinical features was assessed using Spearman’s rank correlation coefficient. 'Adjusted' refers to the correlation coefficients and p-values between variables after controlling for disease duration and iNPHGS total score (non-parametric partial correlation).

iNPHGS: idiopathic Normal Pressure Hydrocephalus Grading Scale, mRS: modified Rankin Scale, TUG: Timed Up and Go test, FBS: Functional Balance Scale, MDS-UPDRS: Movement Disorder Society-Unified Parkinson's Disease Rating Scale, MMSE: Mini Mental State Examination, FAB: Frontal Assessment Battery, TMT-A: Trail Making Test-A, CBT: Counting-backward Test, FES: First error score, REI: Reverse effect index, PVF: Phonemic Verbal Fluency, CVF: Category Verbal Fluency, WMS-R ACI: Wechsler Memory Scale-Revised Attention/Concentration Index, NPI: Neuropsychiatric Inventory.
